# Macular Retinal Ganglion Cell Complex Thickness and Its Relationship to the Optic Nerve Head Topography in Glaucomatous Eyes with Hemifield Defects

**DOI:** 10.1155/2011/914250

**Published:** 2010-11-01

**Authors:** Seiji T. Takagi, Yoshiyuki Kita, Asuka Takeyama, Goji Tomita

**Affiliations:** Department of Ophthalmology, Ohashi Medical Center, Toho University, 2-17-6 Ohashi, Meguro-ku, Tokyo 153-8515, Japan

## Abstract

*Purpose*. To evaluate the relationship between the macular ganglion cell complex (mGCC) thickness, which is the sum of the retinal nerve fiber, ganglion cell, and inner plexiform layers, measured with a spectral-domain optical coherence tomograph and the optic nerve head topography measured with a confocal scanning laser ophthalmoscope in glaucomatous eyes with visual field defects localized predominantly to either hemifield. *Materials and Methods*. The correlation between the mGCC thickness in hemispheres corresponding to hemifields with and without defects (damaged and intact hemispheres, respectively) and the optic nerve head topography corresponding to the respective hemispheres was evaluated in 18 glaucomatous eyes. *Results*. The mGCC thickness was significantly correlated with the rim volume, mean retinal nerve fiber layer thickness, and cross-sectional area of the retinal nerve fiber layer in both the intact and the damaged hemispheres (*P* < .05). *Discussion*. For detecting very early glaucomatous damage of the optic nerve, changes in the thicknesses of the inner retina in the macular area and peripapillary RNFL as well as rim volume changes in the optic nerve head are target parameters that should be carefully monitored.

## 1. Introduction

The macular thickness is reduced in glaucomatous eyes compared with normal eyes [1–3]. This reduction is attributable mainly to the loss of retinal ganglion cells and retinal nerve fibers [3–5]. Using a newly developed software for automatic measurements of macular ganglion cell complex (mGCC) thickness, which is the sum of the thicknesses of the retinal nerve fiber, ganglion cell, and inner plexiform layers, Tan et al. demonstrated that automatic mGCC measurements with a spectral-domain optical coherence tomograph (SD-OCT) have better diagnostic accuracy and repeatability than macular retinal thickness measurements with a time-domain (TD) OCT [6]. On the other hand, topographic measurements of the optic nerve head with a confocal scanning laser ophthalmoscope (CSLO) have demonstrated a high correlation with clinical estimates of the optic nerve by expert assessment at an independent reading center after correcting for the optic disc size [7]. Nowadays, a CSLO is the standard tool to evaluate the optic nerve head topography. However, the relationship between macular structural changes and topographic changes in the optic nerve head in glaucoma is not clear. For combined application of mGCC thickness and optic nerve head topographic measurements to detect glaucoma, the correlation between the optic nerve head topography and the mGCC thickness should be clarified.

In this study, we aimed to elucidate the relationship between the mGCC thickness measured with an automatic measurement algorithm of an SD-OCT and the optic nerve head topography measured with a CSLO in glaucomatous eyes with hemifield defects.

## 2. Subjects and Methods

### 2.1. Subjects and Examinations

Participants were recruited at the Department of Ophthalmology, Toho University Ohashi Medical Center, Tokyo, Japan. The Toho University Ohashi Medical Center Institutional Review Board approved all protocols, and the study adhered to the tenets of the Declaration of Helsinki. The study protocols were thoroughly explained to all participants and their written informed consent was obtained.

All subjects underwent complete ophthalmologic examination, including assessment of medical and family history, visual acuity testing with refraction, slit-lamp biomicroscopy including gonioscopy, intraocular pressure (IOP) measurement with Goldmann applanation tonometry, and dilated stereoscopic fundus examination. Their visual field sensitivity was tested by using Humphrey field analyzer (model 750i, Carl Zeiss Meditec, Inc., Dublin, CA) 30-2 with Swedish interactive threshold algorithm (SITA) standard automated perimetry (SAP). All subjects underwent the Humphrey visual field (HVF), OCT, and CSLO tests within a 3-month time window.

### 2.2. Inclusion and Exclusion Criteria

The inclusion criteria were as follows: normal open anterior chamber angles on slit-lamp biomicroscopic and gonioscopic examinations of both eyes; glaucomatous optic nerve head appearance on stereoscopic evaluation (i.e., focal or generalized narrowing or disappearance of the neuroretinal rim with a vertical cup-to-disc area ratio of >0.7 or retinal nerve fiber layer [RNFL] defects indicating glaucoma) and corresponding visual field abnormalities in either the superior or the inferior hemifield exclusively by repeatable SAP results; and best-corrected visual acuity of ≥15/20 with no media opacities, refractive errors in the spherical equivalent not exceeding −6 or +3 diopters, and cylindrical correction within 3.0 diopters. 

In addition, the subjects had to be familiar with SAP testing from at least two previous visual field examinations and have a reliable HVF with SITA 30-2 standard tests (fixation loss <20%, false-positive and false-negative rates <33%). They were also required to have at least one eye meeting the following criteria for hemifield defects: a cluster of three or more contiguous points in the pattern deviation plot of the HVF with a probability of <5% in either the superior or the inferior hemifield, with at least one point having a probability level of <1%; the opposite hemifield not having a point with a probability level equal to or worse than 2%, or a cluster of three or more contiguous points with a probability of <5%; glaucoma hemifield test results outside the normal limits.

The exclusion criteria were a history of intraocular surgery, presence of other intraocular eye diseases or other diseases affecting the visual fields (e.g., pituitary lesions, demyelinating diseases, diabetic retinopathy), and treatment with medications known to affect visual field sensitivity. If both eyes met all the criteria, one eye was randomly selected.

### 2.3. Measurements of mGCC Thickness

The selected eyes were scanned by using RTVue-100 (Optovue, Inc., Fremont, CA) with software version 2.0.4.0, which uses a scanning laser diode to emit a scan beam with a wavelength of 840 ± 10 nm to provide images of ocular microstructures. In this study, the GCC scanning protocol was used for the mGCC thickness measurements. The GCC protocol is a 7 mm × 7 mm raster scan composed of one horizontal B scan with 800 A scans, and 17 vertical B scans with 934 A scans. The mean GCC thickness of the superior and inferior hemispheres was calculated. A well-trained operator obtained good-quality OCT images with pupillary dilation. The criteria for determining scan quality were signal strength of at least 50 or more (as suggested by the manufacturer), a clear fundus image allowing a foveal pit, even and dense color saturation throughout all retinal layers with red color visible in the retinal pigment epithelium without interruptions, and a continuous scan pattern without missing or blank areas.

### 2.4. Optic Nerve Head Measurements

The parameters of the optic nerve head topography were measured with Heidelberg Retina Tomograph II (HRT-II, software version 3.1.2.4; Heidelberg Engineering GmbH, Heidelberg, Germany) [8–10]. HRT-II uses a diode laser (670-nm wavelength) to scan the retinal surface sequentially in the horizontal and vertical directions at multiple focal planes. By using confocal scanning principles, a three-dimensional topographic image is constructed from a series of optical image sections at consecutive focal planes. The topographic image determined from the acquired three-dimensional image consists of 384 × 384 (147,456) pixels, each of which is a measure of the retinal height at its corresponding location. For every subject in this study, images were obtained through dilated pupils with a 15-degree field of view. 

Three topographic images were obtained, combined, and automatically aligned to create a single mean topographic image for analysis. A contour line of the optic disc margin was drawn around the inner margin of the peripapillary scleral ring by a well-trained operator, while viewing nonstereo color fundus photographs. The contour line was reviewed in the topographic and reflectance images and the height profile graph included in the instrument by the same operator. Twelve HRT-II parameters were analyzed: disc area, cup area, rim area, cup-to-disc area ratio, cup volume, rim volume, mean cup depth, maximum cup depth, height variation contour, cup shape measure, mean RNFL thickness, and RNFL cross-sectional area. Magnification errors were corrected by using the subjective refractive status and corneal curvature measurements. The analysis was restricted to the eyes that had valid optic disc measurements with HRT-II. Good image quality was defined by appropriate focus, brightness, and clarity; minimal eye movement; optic disc centered in the image; a standard deviation (SD) of the mean topographic image less than 50 *μ*m. The eyes for which good-quality images could not be obtained were excluded from the analysis.

### 2.5. Statistical Analysis

The correlation between the mGCC thickness measurements in the hemispheres corresponding to the hemifields with and without visual field defects (damaged and intact hemispheres, resp.) and the HRT-II parameters corresponding to the respective hemispheres (90 degrees superior or inferior to the optic nerve head topography) was evaluated. For example, when an eye had superior hemifield defects, the inferior hemisphere was the damaged hemisphere and the superior hemisphere was the intact hemisphere. In this case, the damaged hemisphere comprised 90 degrees in the inferior optic nerve head topography and the intact hemisphere was 90 degrees in the superior optic nerve head topography.

Statistical analyses were performed by using SPSS version 17.0 (SPSS, Inc., Chicago, IL). Data are presented as the mean ± SD. The two-tailed paired *t*-test was performed to evaluate the difference in the parameters between the damaged and the intact hemispheres. When the mean difference (mean of the difference between individual eyes) is 10 and its SD is 10, or the mean difference is 0.1 and the SD is 0.1, 18 subjects provide 99% power with alpha = 0.05, two tails, in the paired *t*-test. Linear regression analysis and Pearson's correlation coefficients (*R*) were used to assess the relationship between the OCT and the HRT-II parameters. A correlation coefficient of 0.55 has 80% power with alpha = 0.05, with 18 subjects (Fisher Z approximation). A *P* value of < .05 was considered statistically significant.

## 3. Results

In total, 18 eyes with primary open-angle glaucoma (superior hemifield defects: 13 eyes; inferior hemifield defects: five eyes) were studied (Table 1). 

The mGCC thickness in the damaged hemisphere was significantly thinner than that in the intact hemisphere (Table 2). The cup-to-rim area ratio, rim volume, mean RNFL thickness, and RNFL cross-sectional area in the damaged hemisphere were also significantly worse than that in the intact hemisphere (Table 2).

The mGCC thickness was significantly correlated with the cup-to-disc area ratio, rim area, rim volume, mean RNFL thickness, and RNFL cross-section area in the damaged hemisphere. In the intact hemisphere, the mGCC thickness was significantly correlated with the rim volume, mean RNFL thickness, and RNFL cross-sectional area (Table 3).

## 4. Discussion

In this study, after measuring the mGCC thickness by using an SD-OCT and the topographic parameters of the optic nerve head by using a CSLO in glaucomatous eyes with hemifield-localized visual field loss, we found that the mGCC thickness is correlated with the rim volume, mean RNFL thickness, and RNFL cross-sectional area even in the intact hemisphere corresponding to the hemifield without apparent visual field defects.

There are reports of diffuse RNFL damage in eyes with localized visual field abnormalities [11–15]. Grewall et al. reported that the HRT-derived cup-to-disc area ratio is significantly correlated with the mean RNFL thickness in a normal hemifield measured by using an SD-OCT [16]. However, knowledge on the structural changes in the macular area of glaucomatous eyes and their correlation with the optic nerve head topography in a normal visual hemifield is still limited. We found that the diffuse structural damage observed in glaucoma also includes the macular area, particularly the inner retinal structure (GCC thickness). Our observation of the correlation between the mGCC thickness and the RNFL-related HRT-II parameters in the intact hemisphere is reasonable because the mGCC thickness can be considered to represent damage mainly of the ganglion cells and their axons. 

In our study, the cup area-to-disc area ratio and rim area were correlated with the mGCC thickness in the damaged hemisphere but not in the intact hemisphere. The reason for this discrepancy is not clear. Considering the significant correlation in the rim volume in the damaged and intact hemispheres, a three-dimensional parameter such as volume might provide more precise information on ganglion cell damage than a two-dimensional parameter such as area does. Another possible explanation is the influence of the size of the optic nerve head on the cup area-to-disc area ratio. As the cup area is significantly correlated with the size of the optic nerve head, a difference in this size between the damaged and the intact hemispheres could influence the measurement of the cup area. However, in the present study, there was no significant difference in the size of the optic nerve head between the hemispheres.

Regarding the reproducibility of the mGCC thickness measurements, Tan et al. showed good reproducibility by using RTVue [6]. However, our study has several limitations. First, the sample size is small; therefore, although a correlation coefficient of 0.55 has 80% power (alpha = 0.05) with 18 subjects, a significant correlation between parameters with a smaller correlation coefficient can be detected with a larger sample. Second, we used only 90 degrees superior and inferior in the optic nerve head topographic measurements; this may underrepresent the changes seen in the mGCC thickness. However, in the GCC scanning protocol of the RTVue, to cover the peripheral areas most affected by glaucoma, the center of the GCC map is placed at 1 mm temporal to the foveal center for better coverage of the temporal region. Therefore, the mGCC thickness seems to reflect the thickness of the peripapillary RNFL located in the more superior or inferior portion of the optic nerve head rather than just the temporal region. The relationship of the temporal topographic parameters of the optic nerve head with the mGCC thickness should be analyzed by another study. Third, correction for ocular magnification due to refraction, axial length, and camera parameters is unavailable in the current RTVue system, although HRT corrects its magnification errors before analysis. Therefore, in our study, the scanning area for the mGCC thickness measurements might have been affected by refractive errors or axial length of the eye. Recently, Kang et al. reported that after adjusting ocular magnification, the average peripapillary RNFL thickness measured by an SD-OCT has no correlation with the spherical equivalent and only a weak positive correlation with axial length [16]. It is still not clear whether mGCC thickness measurements are influenced by ocular magnification or not. Further study to investigate ocular magnification effects on mGCC measurements is needed.

In conclusion, of the 12 HRT-II parameters assessed in glaucomatous eyes with visual field defects restricted to hemifields, most were not significantly associated with the mGCC thickness. Of the three parameters that showed significant correlations in both the damaged and the intact hemispheres, two are related to the RNFL thickness and only one is an actual topographical measurement of the optic nerve head. Particularly in the intact hemisphere, the mGCC thickness is significantly correlated with the RNFL-related HRT parameters. For detecting very early glaucomatous damage of the optic nerve, changes in the thicknesses of the inner retina in the macular area and peripapillary RNFL as well as rim volume changes in the optic nerve head are target parameters that should be carefully monitored.

## Figures and Tables

**Table 1:** Background data of the patients (*n* = 18).

Male/female		8/10
Age (years)		53.6 ± 14.6
Refraction (diopters)*		−3.75 ± 3.56
Intraocular pressure (mmHg)		14.4 ± 1.8
Humphrey visual field		
Mean deviation (dB)		−6.57 ± 3.91
Pattern standard deviation (dB)		9.35 ± 3.80
Mean of the total deviation (dB)		
Hemifield corresponding to the damaged hemisphere		−10.94 ± 7.24
Hemifield corresponding to the intact hemisphere		−2.70 ± 2.64
Mean of the pattern deviation (dB)		
Hemifield corresponding to the damaged hemisphere		−10.41 ± 6.45
Hemifield corresponding to the intact hemisphere		−2.30 ± 1.96

Values are expressed as the mean + SD. *: spherical equivalents.

**Table 2:** The mGCC thickness and HRT-II parameters of the intact and damaged hemispheres.

Parameters	Damaged hemisphere	Intact hemisphere	*P*
Macular area			
mGCC (*μ*m)	75.67 ± 10.16	85.11 ± 10.03	.005
HRT-II parameters			
Disc area (mm^2^)	0.53 ± 0.13	0.54 ± 0.11	.329
Cup area (mm^2^)	0.29 ± 0.14	0.25 ± 0.12	.205
Rim area (mm^2^)	0.22 ± 0.09	0.27 ± 0.12	.058
Cup area/disc area	0.59 ± 0.17	0.46 ± 0.22	.032
Cup volume (mm^3^)	0.09 ± 0.07	0.08 ± 0.08	.254
Rim volume (mm^3^)	0.05 ± 0.03	0.08 ± 0.05	.009
Mean cup depth (mm)	0.34 ± 0.15	0.38 ± 0.20	.211
Maximum cup depth (mm)	0.66 ± 0.19	0.79 ± 0.46	.153
Height variation contour (mm)	0.34 ± 0.14	0.26 ± 0.11	.068
Cup shape measure	0.04 ± 0.08	0.04 ± 0.15	.811
Mean RNFL thickness (mm)	0.18 ± 0.10	0.31 ± 0.17	.012
RNFL cross-sectional area (mm^2^)	0.21 ± 0.19	0.40 ± 0.22	.005

Values are expressed as the mean + SD; *P* values by paired *t*-test (*n* = 18). mGCC = macular ganglion cell complex, HRT-II = Heidelberg Retina Tomograph II, RNFL = retinal nerve fiber layer.

**Table 3:** Correlations between the mGCC thickness and the HRT-II parameters.

HRT-II parameters	Damaged hemisphere		Intact hemisphere	
	*R*	*P*	*R*	*P*
Disc area	0.15	.561	0.22	.378
Cup area	−0.35	.152	0.003	.990
Rim area	0.58	.013	0.24	.343
Cup area/disc area	−0.56	.013	−0.13	.622
Cup volume	−0.20	.450	0.05	.857
Rim volume	0.64	.004	0.54	.021
Mean cup depth	−0.10	.683	0.26	.294
Maximum cup depth	0.08	.745	0.25	.310
Height variation contour	−0.11	.349	−0.14	.593
Cup shape measure	−0.35	.126	0.16	.520
Mean RNFL thickness	0.58	.012	0.66	.003
RNFL cross-sectional area	0.57	.014	0.70	.001

mGCC = macular ganglion cell complex, HRT-II = Heidelberg Retina Tomograph II, RNFL = retinal nerve fiber layer, *n* = 18.
